# Metabolomic Characterization Reveals Organ-Specific Accumulation of Secondary Metabolites in Petiole Glands of *Idesia polycarpa*

**DOI:** 10.3390/metabo16090615

**Published:** 2026-08-27

**Authors:** Fangming Liu, Xingyue Xiong, Yanmei Wang, Li Dai, Zhi Li, Xiaodong Geng, Juan Wang, Yongyu Ren, Qifei Cai, Zhen Liu

**Affiliations:** 1 Henan Province Engineering Technology Research Center for *Idesia*, Zhengzhou 450046, China; fangmingliu-0930@henau.edu.cn (F.L.); wym@henau.edu.cn (Y.W.); daili@henau.edu.cn (L.D.); lizhi@henau.edu.cn (Z.L.); xiaodonggeng@163.com (X.G.); wangjuan475@163.com (J.W.); yongyuren_1128@henau.edu.cn (Y.R.); 2 National Forestry and Grassland Administration Key Laboratory for Central Plains Forest Resources Cultivation, Zhengzhou 450046, China; 3 College of Forestry, Henan Agricultural University, Zhengzhou 450046, China; 4 School of Forest Resources and Environmental Science, Michigan Technological University, Houghton, MI 49931, USA; 5 Henan Academy of Forestry Sciences, Zhengzhou 450008, China; xinyueing45@163.com

**Keywords:** *Idesia polycarpa*, petiole gland, widely targeted metabolomics, secondary metabolites, organ-specific accumulation, plant specialized metabolism

## Abstract

**Highlights:**

**What are the main findings?**

- Ultra-performance liquid chromatography-tandem mass spectrometry (UPLC-MS/MS)-based widely targeted metabolomics revealed distinct organ-specific accumulation patterns of secondary metabolites in *Idesia polycarpa* petiole glands.
- Petiole glands exhibited the greatest metabolic diversity among examined organs and contained 39 exclusively detected metabolites, including flavonoids, proanthocyanidins, and phenolic acid derivatives.

**What are the implications of the main findings?**

- The secondary metabolite profile of petiole glands suggests their potential ecological significance in plant–environment interactions.
- This study establishes the first metabolomic baseline for *I. polycarpa* petiole glands and provides a valuable resource for future studies on gland function and bioactive metabolites.

**Abstract:**

Background/Objectives: *Idesia polycarpa* Maxim. possesses conspicuous glandular protuberances on its petioles, but the chemical composition and potential biological significance of these structures remain largely unexplored. This study aimed to characterize the metabolic profile of *I. polycarpa* petiole glands and to determine their organ-specific accumulation patterns of secondary metabolites compared with leaves and shoot tips. Methods: A widely targeted metabolomics approach based on ultra-performance liquid chromatography tandem mass spectrometry (UPLC-MS/MS) was applied to analyze petiole glands, leaves, and shoot tips collected from the same *I. polycarpa* individuals. Multivariate statistical analyses and differential metabolite profiling were performed to compare metabolic characteristics among the three organs. Results: Petiole glands contained 700 detected metabolites, representing the highest metabolic diversity among the three examined organs, and 39 metabolites were exclusively detected in petiole glands. Principal component analysis clearly separated petiole glands from leaves and shoot tips, demonstrating their distinct metabolic profile. Compared with other organs, petiole glands showed preferential accumulation of several secondary metabolite classes, including flavonoids, tannins, alkaloids, and phenolic acids. The gland-exclusive metabolites included flavonoids such as kaempferide, kaempferitrin, and tectoridin, as well as diverse proanthocyanidins and phenolic acid derivatives. Conclusions: This study provides the first systematic metabolomic characterization of *I. polycarpa* petiole glands and reveals their organ-specific accumulation of secondary metabolites. The unique metabolic profile of petiole glands suggests potential ecological relevance and provides a foundation for further investigations into the biological functions of gland-associated metabolites.

## 1. Introduction

Plant secretory structures—including glandular trichomes, resin ducts, laticifers, and extrafloral nectaries (EFNs)—are among the most functionally diverse organs in the plant kingdom. Although they represent a small fraction of total plant biomass, these structures are disproportionately important in mediating plant–environment interactions. EFNs and other petiole- or leaf-borne glands are found in over 100 plant families and function primarily in indirect defense: by secreting nectar or other chemical rewards, they recruit predatory ants and parasitoid insects that protect the plant against herbivory [1,2]. Beyond indirect defense, glandular structures are also known to accumulate high concentrations of direct-defense chemicals—tannins, alkaloids, and phenolic acids—that deter or intoxicate feeding insects [3,4]. The secondary metabolite composition of secretory structures is often organ-specific: glandular tissues frequently contain compound classes that are largely absent from adjacent non-glandular organs such as leaves or stems [4,5]. Understanding the metabolic identity of plant glands is therefore central to understanding how plants deploy their chemical defenses in space.

The Salicaceae—a family that includes Populus (poplars), Salix (willows), and the more broadly circumscribed tropical and subtropical genera—have been a model system for studying plant secondary metabolites in a defense context. Phenolic glycosides structurally derived from salicyl alcohol (‘salicinoids’) are the signature defensive metabolites of this family, accumulating to up to 30% of dry mass in leaves and bark of Populus and Salix, and exerting well-documented negative effects on the performance of generalist herbivores [6,7]. Condensed tannins (proanthocyanidins) and flavonoids represent additional layers of chemical defense in Salicaceae, with their accumulation regulated by salicylic acid signaling and induced by herbivore attack [8,9]. Several Salicaceae genera also bear petiole- or leaf-borne glandular structures morphologically similar to EFNs. In *Passiflora* (Passifloraceae, closely allied to Salicaceae in the Angiosperm Phylogeny Group (APG) system), petiolar EFNs are particularly well studied and serve as the interface for ant–plant mutualisms [2]. Within the core Salicaceae, petiolar and leaf-base glands are present in multiple genera, but their chemical composition has rarely been systematically investigated at the metabolome level.

**Figure 1:**
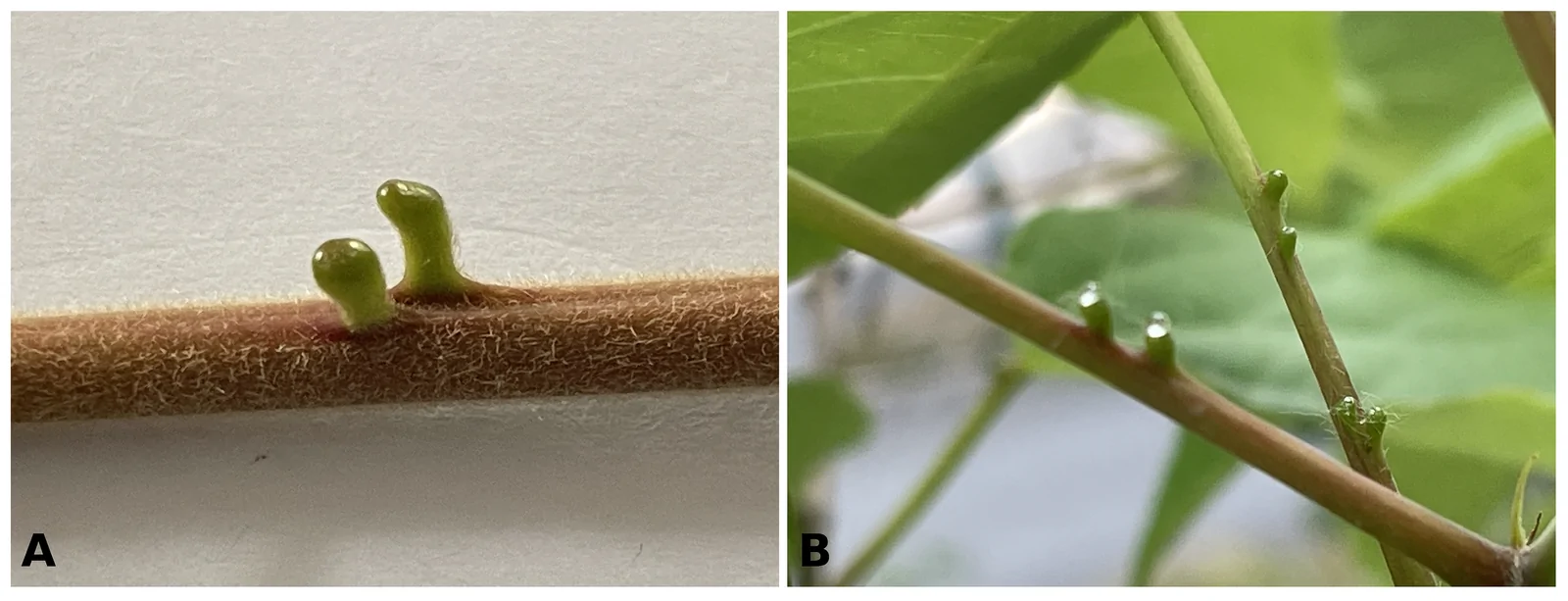
Petiole glandular structures of *I. polycarpa*. (**A**) Close-up morphology of paired glandular structures at a petiolar node. (**B**) Visible liquid droplets accumulating at gland apices under field conditions, confirming active secretion. Panel (**A**) adapted from Figure 2a and panel (**B**) adapted from Figure 3a of Liu et al. [15], distributed under the terms of the CC BY 4.0 license.

*Idesia polycarpa* Maxim. is a deciduous tree in the Salicaceae (formerly Flacourtiaceae) widely distributed across the warm-temperate and subtropical zones of East Asia [10,11]. It is valued primarily as a woody oil crop—its fruits contain over 30% oil rich in linoleic acid—but also as a timber and ornamental species [12,13]. The recent availability of a chromosome-level reference genome further establishes *I. polycarpa* as a tractable system for investigating organ-specific specialized metabolism [14]. Across its vegetative body, *I. polycarpa* bears conspicuous glandular protuberances on the petioles of every leaf. These structures—elongated, globular, or oblate in form—are distributed at the basal, middle, and apical regions of each petiole. They develop synchronously with leaf expansion, progressively darken with leaf maturation, and senesce before leaf abscission. These structures are illustrated in Figure 1 and have been documented to actively secrete liquid under field conditions [15].

Such petiole-borne glands are among the most visually distinctive features of *I. polycarpa* vegetative morphology, yet nothing is known about their chemical composition. Previous phytochemical investigations of *I. polycarpa* have focused on fruits and, to a lesser extent, leaves, identifying phenolic glycosides (Idescarpin, Salirepin, Idesin, Idesolide) with antioxidant, anti-inflammatory, and anti-adipogenic activities [16,17,18,19], and demonstrating that leaf phenolics are toxic to lepidopteran larvae [20]. However, the petiole gland—the most morphologically conspicuous glandular structure of this species—has never been the subject of chemical investigation of any kind.

The metabolic composition of *I. polycarpa* vegetative organs has recently begun to be characterized. Li et al. [21] employed UPLC-MS/MS (ultra-performance liquid chromatography tandem mass spectrometry)-based widely targeted metabolomics to compare the metabolic profiles of leaves and shoot tips, identifying 378 differential metabolites and establishing that leaves are enriched in secondary metabolites while shoot tips accumulate more primary metabolites. That study left the petiole gland entirely uncharacterized. The present study extends this work by providing, to the best of our knowledge, the first systematic metabolomics characterization of *I. polycarpa* petiole glands, and the first metabolomic characterization of a petiole gland reported in the Salicaceae. By comparing the gland metabolome with those of leaves and shoot tips collected from the same trees at the same time, we address three specific questions: (1) What is the full metabolite inventory of *I. polycarpa* petiole glands? (2) Which metabolites are enriched in or exclusive to petiole glands relative to other vegetative organs? (3) What do these patterns suggest about the ecological role of petiole glands in this species? Whereas Li et al. [21] addressed only the leaf-shoot tip comparison, the present study introduces an entirely new organ type—the petiole gland—and encompasses statistical analyses, including three-way principal component analysis, Venn diagram comparisons, and pairwise differential analyses involving the gland, that were not performed in that prior work.

## 2. Materials and Methods

### 2.1. Plant Material and Sample Collection

Petiole glands, leaves, and shoot tips of *I. polycarpa* were collected concurrently in July 2020 from trees grown at the Forestry Experimental Station of Henan Agricultural University, Zhengzhou, China (113°38′ E, 34°47′ N). Mature leaves were excised from the mid-canopy region of the main stem. Sampling was restricted to the mid-canopy region because canopy position and plant height are known to substantially influence secondary metabolite concentrations in woody Salicaceae and related species [22,23]; fixing sampling position across all three organs was intended to minimize this source of variability and ensure comparability among organ types. Shoot tips were harvested from the terminal portions of lateral branches at mid-canopy height, with any attached young leaves removed. Petiole glands of elongated, globular, and oblate morphology were pooled from multiple petioles and designated as the study material. At the time of collection (July), all petiole glands were at the mature developmental stage, as evidenced by their dark pigmentation and the presence of visible liquid droplets at the gland apices, confirming active secretion (consistent with field observations documented in Liu et al. [15], Figure 3a). Samples were designated as group A (leaves), B (shoot tips), and C (petiole glands), with three independent biological replicates prepared for each organ (*n* = 3 per group; further detailed below). Immediately after harvest, all samples were flash-frozen in liquid nitrogen for 3 min and stored at −80 °C until analysis. Each biological replicate was prepared by pooling tissues from five randomly selected individual trees at the study site, with branches of varying orientation sampled within each tree to minimize positional bias. The three biological replicates therefore represent three independent pooled samples, each derived from a distinct set of five trees, rather than single trees or single branches. This pooling strategy reduces noise from inter-individual variation but does not permit assessment of tree-to-tree metabolic variability; this limitation is acknowledged in Section 4.6. The protocol followed the same collection strategy as the companion study [21]. Leaves, shoot tips, and petiole glands were originally collected together in a single sampling event and analyzed within a single combined UPLC–MS/MS analytical batch. Leaf and shoot-tip data from this dataset were subsequently reported separately in a companion study [21]; the present study analyzes the complete three-organ dataset, including petiole glands, which were not addressed in that prior work. All cross-organ comparisons reported here are therefore based on data acquired under identical instrumental conditions within a single analytical run. Here, leaves and shoot tips were included as reference organs to enable a comprehensive assessment of petiole gland metabolic specialization. All statistical analyses presented here—including the three-way principal component analysis (PCA), Venn diagram analysis, pairwise orthogonal partial least squares discriminant analysis (OPLS-DA) comparisons involving petiole glands, and hierarchical clustering—were conducted specifically for this study and were not included in Li et al. [21], which examined only the leaf–shoot tip comparison.

### 2.2. Sample Preparation

For each biological replicate, pooled frozen tissue (Section 2.1) was lyophilized and ground together as a single composite sample to a uniform fine powder using a mixer mill (MM 400, Retsch, Haan, Germany; 30 Hz, 1.5 min); tissue from individual trees was not lyophilized or ground separately prior to pooling. All chromatographic-grade solvents (methanol, acetonitrile, and formic acid) were purchased from Thermo Fisher Scientific (Fair Lawn, NJ, USA). Ultrapure water was generated using a Milli-Q purification system (Millipore, Bedford, MA, USA). Approximately 100 mg of each powder was dissolved in 1.2 mL of 70% (*v*/*v*) aqueous methanol, stored at 4 °C overnight, and vortexed six times to enhance solubilization. Extracts were centrifuged at 12,000 rpm for 10 min, and supernatants were filtered through 0.22 µm microporous membranes (Jinteng, Tianjin, China) prior to analysis. Quality control (QC) samples were prepared by pooling equal aliquots from all extracts across the three organ groups (A, B, and C); one QC injection was inserted after every 10 analytical samples within this single batch to monitor instrumental stability and analytical reproducibility across the full sample set.

### 2.3. UPLC-MS/MS Analysis

Chromatographic separation was performed on a SHIMADZU Nexera X2 UPLC system (Shimadzu, Kyoto, Japan) with an SB-C18 column (1.8 µm, 2.1 mm × 100 mm; Agilent Technologies, Santa Clara, CA, USA). Mobile phases were 0.1% (*v*/*v*) formic acid in water (A) and 0.1% (*v*/*v*) formic acid in acetonitrile (B). Gradient conditions: 0–9.0 min, 5 → 95% B; 9.0–10.0 min, 95% B; 10.0–11.1 min, 95 → 5% B; 11.1–14.0 min, 5% B. Flow rate: 0.35 mL·min^−1^; column temperature: 40 °C; injection volume: 4 µL. Mass spectrometric detection was performed on an Applied Biosystems 4500 QTRAP (AB Sciex, Framingham, MA, USA) with electrospray ionization (ESI) in positive and negative modes. Source temperature: 550 °C; ion spray voltage: +5500 V (positive)/−4500 V (negative); curtain gas: 25 psi. Metabolites were quantified by multiple reaction monitoring (MRM) and annotated by matching precursor *m*/*z*, fragment ions, and relative intensity patterns against Metware’s self-built spectral database (MWDB, Metware Database, Wuhan Metware Biotechnology Co., Ltd., Wuhan, China), generated using Metware’s Widely-Targeted Metabolomics platform. The MWDB organizes reference spectra internally by compound class (see Table 1 for the complete category-level breakdown, including flavonoids, phenolic acids, lipids, amino acid derivatives, organic acids, nucleotide derivatives, tannins, alkaloids, lignins/coumarins, and terpenoids). Metware’s identification criteria comprise two confidence levels: Level A requires matching of the full MS/MS spectrum and RT to the database entry, while Level B is based on matching of precursor ion (Q1), fragment ion (Q3), RT, DP, and CE alone. Neither level involves authentic standards analyzed alongside the samples; Level A corresponds approximately to MSI Level 2 (putatively annotated), and Level B is closer to MSI Level 3 (putatively characterized compound class). Annotations for compounds not independently verified against authentic standards—including the proanthocyanidins and select flavonoids highlighted in Section 4—should be regarded as putative.

### 2.4. Data Processing and Statistical Analysis

Chromatographic peaks were integrated and corrected using MultiQuant (v1.6.3; AB Sciex). Values below the detection limit were treated as missing values and excluded from statistical analysis. After unit variance (UV) scaling, PCA was performed to visualize global metabolic variation and evaluate biological replicate consistency. Pairwise orthogonal partial least squares discriminant analysis (OPLS-DA) was conducted to identify differential metabolites between organ pairs; metabolites were considered significantly differential when variable importance in projection (VIP) ≥ 1 and fold change (FC) ≥ 2 or ≤0.5. For reference, metabolites meeting the VIP ≥ 1 threshold corresponded to |p(corr) [1]| values of 0.84–1.00 (A vs. C) and 0.81–1.00 (B vs. C) in the underlying OPLS-DA S-plots, confirming strong concordance between VIP-based selection and correlation magnitude. Model quality and robustness were assessed using *R*^2^*X*, *R*^2^*Y*, and *Q*^2^ statistics together with 200-permutation tests. OPLS-DA results were interpreted in conjunction with PCA and metabolite abundance patterns because of the limited biological replication (*n* = 3). KEGG (Kyoto Encyclopedia of Genes and Genomes) pathway mapping of differential metabolites was performed by Wuhan Metware Biotechnology Co., Ltd. (Wuhan, China) using a hypergeometric test against the KEGG-annotated subset of the metabolome (260 of 728 total metabolites, 36%), with pathway significance evaluated using both uncorrected *p*-values and Benjamini–Hochberg-corrected *p*-values. Hierarchical clustering heatmaps of differential metabolites were generated by Wuhan Metware Biotechnology Co., Ltd. (Wuhan, China) using the MWDB platform, based on Z-score normalized relative abundance. All other multivariate analyses, Venn diagrams, and volcano plots were generated in R (v4.3.2). Throughout this manuscript, metabolites are described as ‘exclusive’ or ‘unique’ to an organ if they were detected (non-floor value) in all three biological replicates of that organ and in none of the replicates of the other two organs (verified for every reported organ-exclusive metabolite; see Data S1), under the analytical conditions and detection limits of the present widely targeted metabolomics platform (*n* = 3 per organ); this operational definition does not preclude the possibility that these compounds occur at concentrations below the limit of detection in the other organs.

## 3. Results

### 3.1. Petiole Glands Harbor the Richest and Most Distinctive Metabolome of the Three Organs

**Figure 2:**
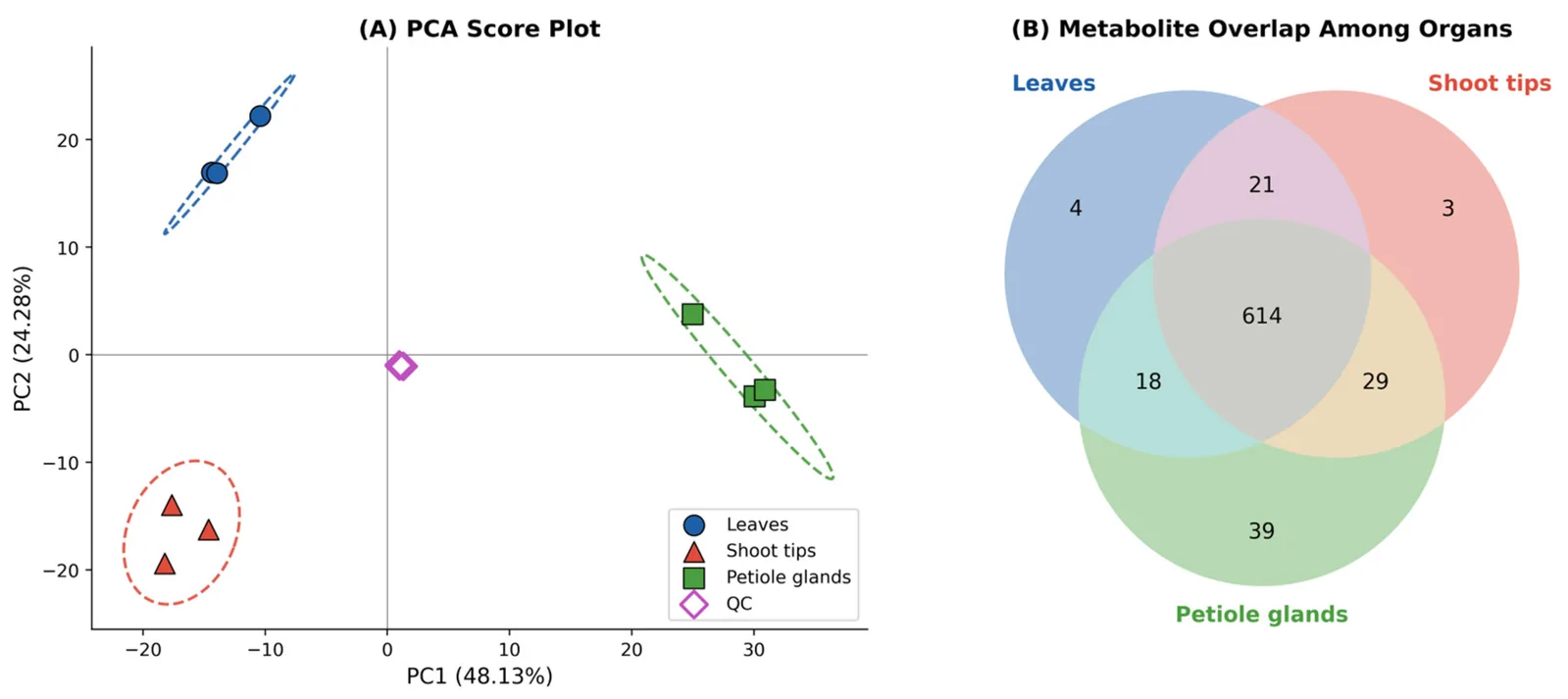
Metabolic overview of three vegetative organs of *Idesia polycarpa*. (**A**) Principal component analysis (PCA) score plot based on all 728 detected metabolites. Each point represents one biological replicate (*n* = 3 per group). Dashed ellipses indicate 95% confidence intervals. (**B**) Venn diagram showing the number of metabolites shared among and exclusive to leaves, shoot tips, and petiole glands. Leaf and shoot-tip biological samples originate from the single collection and analytical batch described in the companion study [21].

**Figure 3:**
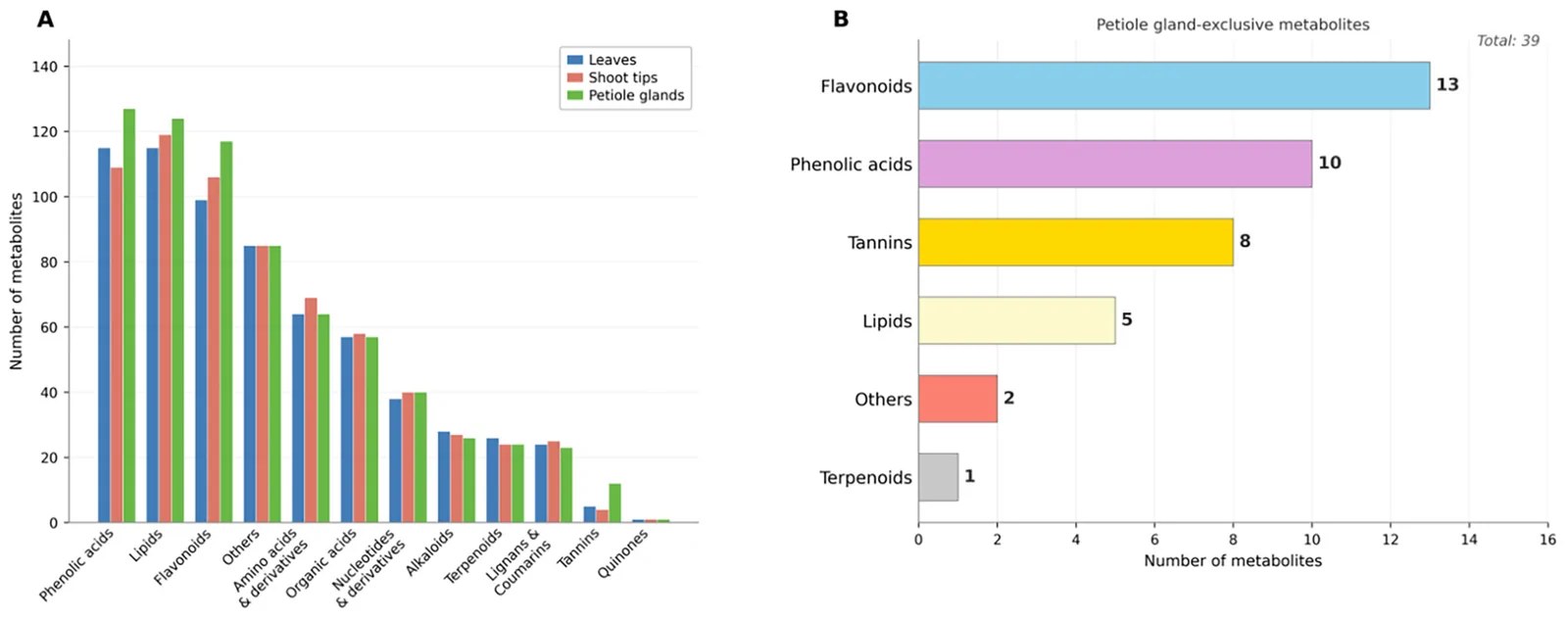
Metabolite composition of *Idesia polycarpa* petiole glands in comparison with leaves and shoot tips. (**A**) Number of metabolites detected in each compound class across the three organs. Petiole glands showed the highest metabolite richness in phenolic acids (127), flavonoids (117), and tannins (12) relative to leaves and shoot tips. (**B**) Classification of 39 metabolites detected exclusively in petiole glands (total: 39), comprising flavonoids (*n* = 13), phenolic acids (*n* = 10), tannins (*n* = 8), lipids (*n* = 5), others (*n* = 2), and terpenoids (*n* = 1). Colors in (**B**) correspond to compound classes shown in (**A**).

UPLC-MS/MS analysis identified a total of 728 metabolites across the three organs of *I. polycarpa* (Table 1). Among the three organs, petiole glands contained the greatest number of metabolites (700 compounds), followed by shoot tips (667) and leaves (657). Of the 728 total metabolites, 614 were shared among all three organs. Thirty-nine metabolites were detected exclusively in petiole glands—nearly ten times the number unique to leaves (4) or shoot tips (3) (Figure 2B). Of the remaining 68 metabolites shared by exactly two organs, the largest subset (29) was shared between shoot tips and petiole glands, while only 18 were shared between leaves and petiole glands and 21 between leaves and shoot tips—indicating that petiole glands’ metabolic distinctiveness arises primarily from organ-exclusive compounds rather than partial overlap with either of the other two organs. Flavonoids (120 compounds total) and phenolic acids (130 compounds total) dominated the secondary metabolome across all three organs, while lipids (127), amino acid derivatives (70), organic acids (58), and nucleotide derivatives (40) were the most abundant primary metabolite classes. Petiole glands showed the highest metabolite richness in phenolic acids (127 vs. 115 in leaves and 109 in shoot tips), flavonoids (117 vs. 99 and 106), and tannins (12 vs. 5 and 4) (Figure 3A).

### 3.2. PCA Separates Petiole Glands from Leaves and Shoot Tips

PCA of all 728 metabolites revealed a clear, three-way separation of the organs (Figure 2A). PC1 explained 48.13% of total variance and PC2 explained 24.28% (cumulative: 72.41%). Petiole glands were the most distinctly separated group, displaced along PC1 from both leaves and shoot tips, which clustered more closely together—consistent with their shared context as above-ground photosynthetic and meristematic tissues. Biological replicates clustered tightly within each organ group, and QC samples co-clustered at the centroid of the score plot, confirming high analytical reproducibility throughout. The pronounced displacement of petiole glands along PC1 indicates that their metabolic composition is fundamentally distinct from both other vegetative organs—a pattern that motivates the detailed class-by-class analysis presented below.

### 3.3. Petiole Glands Are Enriched in Secondary Metabolites Relative to Leaves and Shoot Tips

**Figure 4:**
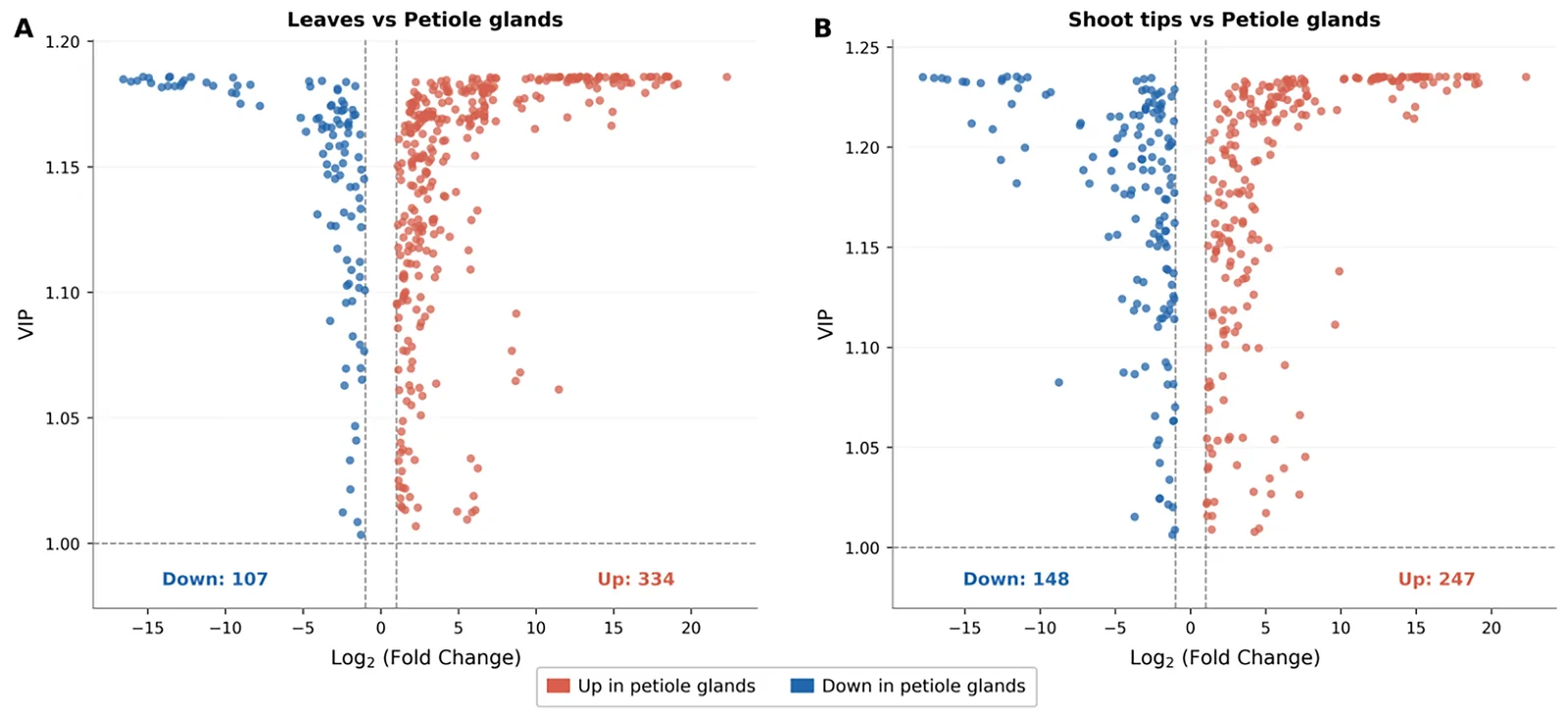
Volcano plots of differential metabolites between petiole glands and other organs. (**A**) Leaves vs. petiole glands; (**B**) Shoot tips vs. petiole glands. Red dots indicate metabolites significantly upregulated in petiole glands; blue dots indicate metabolites significantly downregulated in petiole glands. Dashed lines indicate thresholds of variable importance in projection (VIP) = 1 and Log2(fold change) = ±1.

Pairwise OPLS-DA identified large numbers of differential metabolites in both comparisons involving petiole glands (Figure 4; Table 2). Between leaves and petiole glands (A vs. C), 441 differential metabolites were identified from 725 co-detected compounds: 334 were upregulated in petiole glands (including 84 lipids, 72 flavonoids, 55 phenolic acids, 14 alkaloids, 8 tannins, 8 lignins/coumarins), while 107 were more abundant in leaves (dominated by 30 phenolic acids and 19 flavonoids). Between shoot tips and petiole glands (B vs. C), 395 differential metabolites were identified from 724 co-detected compounds: 247 were upregulated in petiole glands (including 56 lipids, 58 flavonoids, 46 phenolic acids, 11 alkaloids, 10 tannins, 11 lignins/coumarins), while 148 were more abundant in shoot tips—predominantly primary metabolites including 21 nucleotide derivatives, 20 amino acid derivatives, and 16 organic acids. In both pairwise comparisons, the metabolites enriched in petiole glands were dominated by flavonoid, tannin, and alkaloid classes—compound classes reported to contribute to plant defense in other systems, though not all secondary metabolites serve this role—while metabolites enriched in the other organs were predominantly primary metabolites [21]. Compound-specific evidence bearing on a candidate defensive role for petiole gland-enriched metabolites is presented in Section 4. Both OPLS-DA models exhibited excellent goodness-of-fit and predictive ability (A vs. C: *R*^2^*X* = 0.793, *R*^2^*Y* = 1.00, *Q*^2^ = 0.987; B vs. C: *R*^2^*X* = 0.766, *R*^2^*Y* = 1.00, *Q*^2^ = 0.984), and 200-permutation tests confirmed that neither model was overfit (*p* < 0.005 for both *R*^2^*Y* and *Q*^2^ in each comparison; Figure S1).

### 3.4. Hierarchical Clustering Confirms Enrichment of Defense-Associated Secondary Metabolite Classes in Petiole Glands

**Figure 5:**
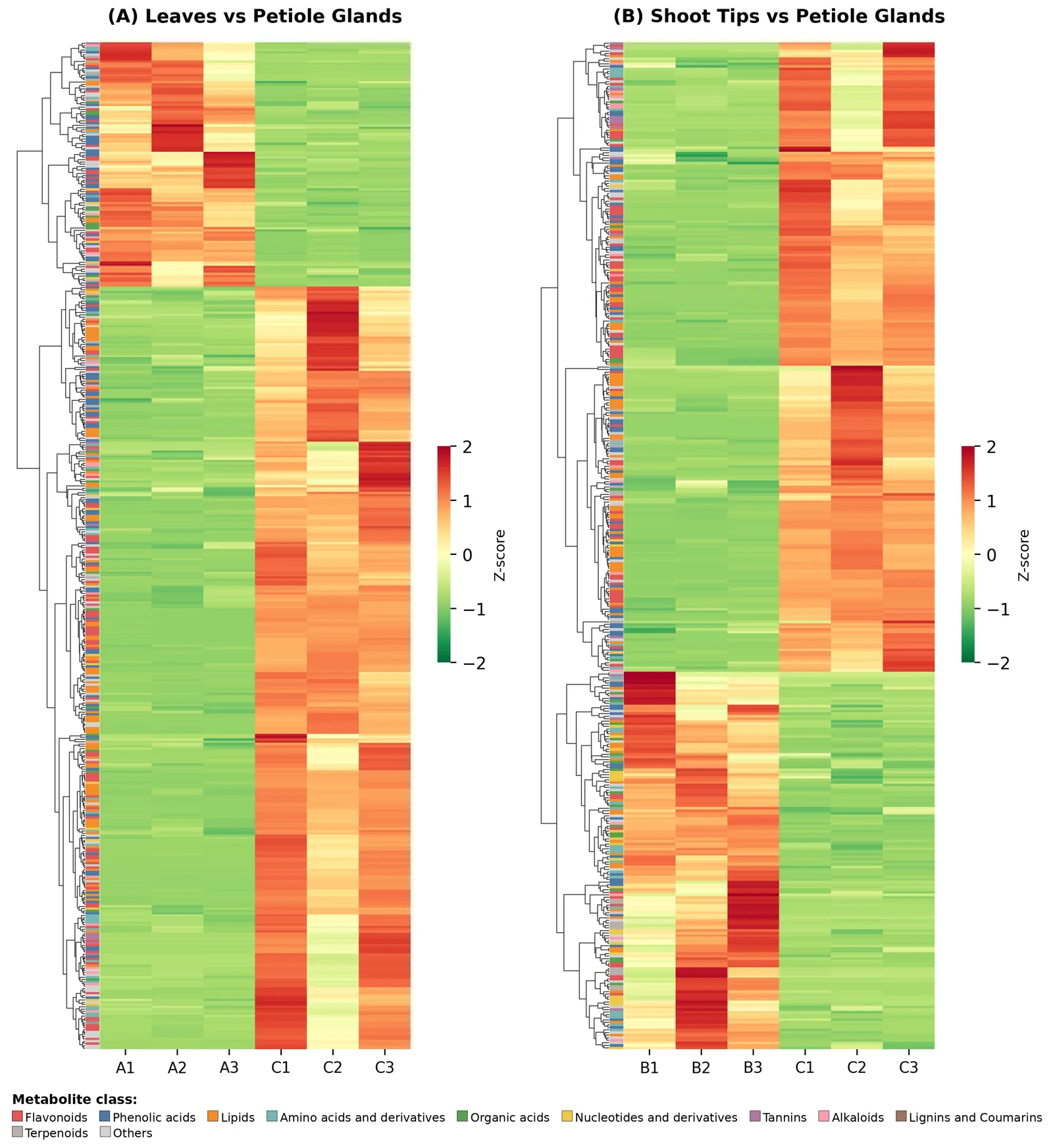
Hierarchical clustering heatmap of differential metabolites between petiole glands and other organs of Idesia polycarpa, shown on a unified color scale. (**A**) Leaves vs. petiole glands (441 differential metabolites); (**B**) Shoot tips vs. petiole glands (395 differential metabolites). Each row represents one differential metabolite (independently clustered within each panel) and each column represents one biological replicate (*n* = 3 per group). Color indicates Z-score normalized relative abundance (unit variance scaling); red, high abundance; green, low abundance. Both panels use the same color scale (−2 to 2) to allow direct visual comparison. The color bar on the left of each heatmap indicates metabolite class (see legend). Differential metabolites were identified based on VIP ≥ 1 and fold change ≥2 or ≤0.5.

To further characterize the enrichment patterns, hierarchical clustering was performed on all differential metabolites grouped by chemical class (Figure 5). The results were consistent across classes. Among 100 differential flavonoids, 72 showed peak relative abundance in petiole glands, including 35 of 40 flavonols, all 5 flavanols, all 3 anthocyanins, and all 3 dihydroflavonols. Among 107 differential phenolic acids, 57 were highest in petiole glands versus 28 in leaves and 22 in shoot tips. Among 105 differential lipids, 76 peaked in petiole glands. The tannin class showed the most extreme gland-specific enrichment: 10 of 13 differential tannins—including all 8 proanthocyanidins detected in the study—were most abundant in petiole glands, with none peaking in leaves. Among 25 differential alkaloids, 15 (60%) were highest in petiole glands. In contrast, the enrichment pattern for primary metabolites was reversed: 34 of 56 differential amino acid derivatives and 26 of 35 differential nucleotide derivatives peaked in shoot tips, a pattern consistent with the meristematic, growth-associated character of that tissue [21]. We note, however, that amino acid and nucleotide derivatives are functionally diverse and are not exclusively markers of growth and development; several compounds within these classes also participate in signaling, osmotic adjustment, and stress responses, so this enrichment pattern should not be interpreted as evidence of growth activity alone.

KEGG pathway mapping of the differential metabolite sets further identified tryptophan metabolism as the nominally top-ranked pathway in both organ comparisons (uncorrected *p* < 0.01 in each case; Figure S2; Table S1), though no pathway remained significant after Benjamini–Hochberg correction, reflecting the limited KEGG annotation coverage of the gland-enriched compound classes central to this study (36% overall; 0% for tannins). This result is reported here as an exploratory observation rather than a statistically confirmed pathway enrichment.

### 3.5. Thirty-Nine Metabolites Were Exclusively Detected in Petiole Glands

The 39 metabolites exclusive to petiole glands (operationally defined as detected in petiole glands but not in leaves or shoot tips under the analytical conditions of this study; see Section 2.4) comprised 13 flavonoids, 10 phenolic acid derivatives, 8 tannins (all proanthocyanidins), 5 lipids (lysophospholipids, including one odd-chain species; see Table 3 note), 2 others, and 1 terpenoid (Figure 3B; Table 3). None of these compounds was detected in leaves or shoot tips. Among the unique flavonoids, kaempferide, kaempferitrin, tectoridin, and dihydrokaempferide had well-documented pharmacological activities (see Section 4). The 8 unique proanthocyanidins—procyanidins A2, B1, B2, B3, B4, C1, C2, and A3—represent the complete structural diversity of condensed tannins detected in this study. The 10 unique phenolic acids included galloylated and acylated derivatives: 3,4-digalloylshikimic acid, caffeoylnicotinoyl tartaric acid, and multiple feruloylquinic acid glucosides. For comparison, leaves harbored only 4 unique metabolites (glucosylsyringate, 4-aminoindole, δ-amyrenone, α-amyrenone) and shoot tips only 3 (luteolin-7-O-(6″-caffeoyl)rhamnoside, limettin, S-methylglutathione). The identification confidence levels for these gland-exclusive metabolites are provided in Table 3; all 39 compounds were classified as Level B (putatively characterized compound class, no authentic standard; approximately MSI Level 3), pending confirmation with authentic standards.

### 3.6. Key Shared Metabolites Show Organ-Specific Distribution Patterns

**Figure 6:**
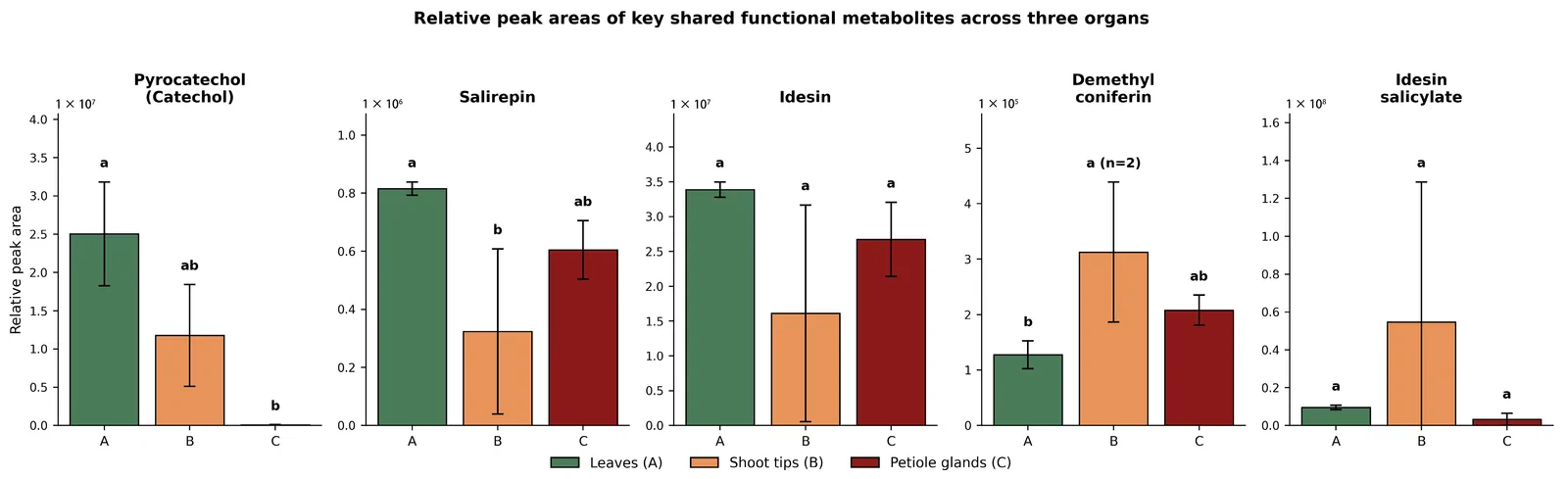
Relative peak areas of key shared functional metabolites across three organs of *I. polycarpa*. These five compounds were first identified as showing differential accumulation between leaves and shoot tips in a companion study [21], which reported their absolute concentrations (µg/g); the relative peak-area data shown here extend that comparison to include petiole glands. Bars show mean relative peak area (*n* = 3 biological replicates, except demethyl coniferin in shoot tips, *n* = 2); error bars indicate SD. Letters above bars indicate statistical grouping; values sharing the same superscript letter are not significantly different (Tukey’s HSD, *p* < 0.05). A, leaves; B, shoot tips; C, petiole glands.

Among shared metabolites with known bioactivities, five compounds showed significant inter-organ differences (Figure 6; Supplementary Table S2). Pyrocatechol (catechol) was most abundant in leaves (relative peak area: 25,037,667), with much lower levels in shoot tips (11,767,200) and petiole glands (74,995). Salirepin was similarly highest in leaves (815,420). Idesin was present in all three organs but peaked in leaves (33,871,667). Demethyl coniferin and idesin salicylate were most abundant in shoot tips (208,440 and 54,724,467, respectively). None of these five compounds showed peak abundance in petiole glands. This result reinforces the conclusion that the distinctive feature of petiole glands is not the shared metabolite pool—where leaves and shoot tips dominate—but rather the 39 gland-exclusive compounds and the disproportionate accumulation of tannin and alkaloid classes documented in Section 3.3 and Section 3.4. Several phenolic compounds, including idesin salicylate (coefficient of variation ≈ 135%), showed disproportionately high inter-individual variability specifically in shoot tips relative to leaves and petiole glands, consistent with greater biological heterogeneity among actively growing meristematic tissue.

## 4. Discussion

### 4.1. Petiole Glands of I. polycarpa Are Metabolically Specialized Glandular Structures

The central finding of this study is that petiole glands of *I. polycarpa* possess a metabolic composition that is both quantitatively richer and compositionally distinct from that of the leaves and shoot tips of the same plant. Glandular or secretory structures in many plant species are known to accumulate distinct secondary metabolite complements relative to adjacent non-glandular tissue [4,5,29], but demonstrating this experimentally requires systematic metabolomics across multiple organs—which the present study provides for *I. polycarpa*, and to the best of our knowledge, represents one of the first metabolomic demonstrations of organ-specific metabolic specialization in a glandular structure within the Salicaceae.

This pattern is supported by the PCA result, in which petiole glands are the most displaced group along PC1 (48.13% of variance), separated from both leaves and shoot tips, while PC2 (24.28%) differentiates leaves from shoot tips. Viewed against the broader literature on plant organ-specific metabolomics [5,30], the gland-specific enrichment documented here—including 39 exclusive metabolites and disproportionate enrichment of several defense-associated metabolite classes (Section 4.2)—is consistent with the petiole gland serving as a potential site of accumulation and/or storage for these compound classes. However, direct functional confirmation (e.g., bioassays or induction experiments) awaits further study. Whether these compounds are actively secreted to the gland surface or accumulated intracellularly cannot be determined from metabolomic data alone, and awaits the histochemical and ultrastructural characterization proposed in Section 4.4.

### 4.2. Pathway-Level Perspective: Terminal Flavonoid Branches Are Preferentially Diverted to Petiole Glands

**Figure 7:**
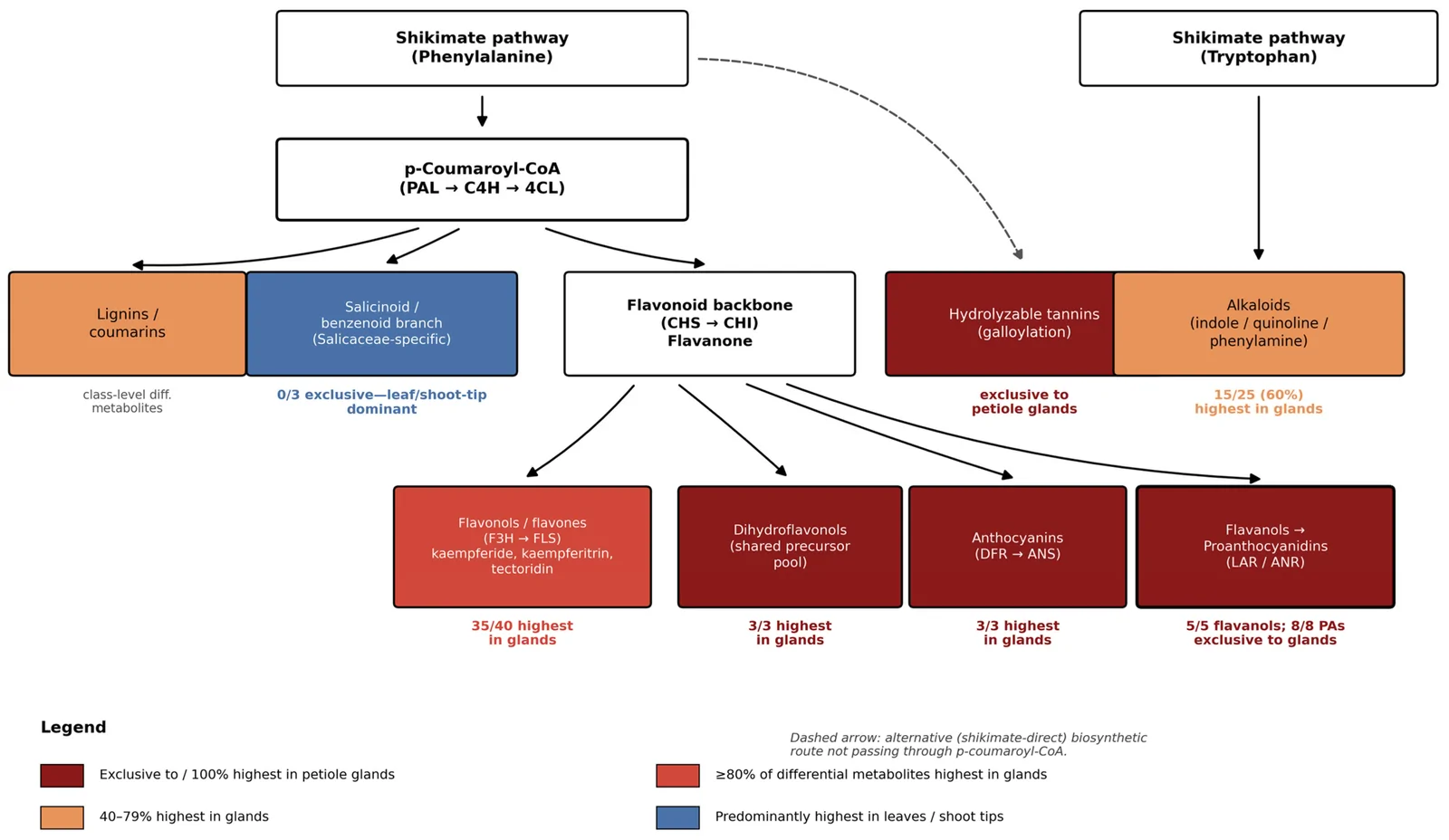
Phenylpropanoid pathway resolution of organ-specific metabolite enrichment in *I. polycarpa* petiole glands. Box color indicates the proportion of differential metabolites in each pathway branch showing peak abundance in petiole glands (see legend). Ratios below each box are drawn from Section 3.3, Section 3.4 and Section 3.5. The dashed arrow indicates the shikimate-derived route to hydrolyzable tannins, which does not pass through the central *p*-coumaroyl-CoA branch point. CHS, chalcone synthase; CHI, chalcone isomerase; F3H, flavanone 3-hydroxylase; FLS, flavonol synthase; DFR, dihydroflavonol reductase; ANS, anthocyanidin synthase; LAR, leucoanthocyanidin reductase; ANR, anthocyanidin reductase.

This pattern is summarized in Figure 7, which maps the organ-specific enrichment ratios reported in Section 3.4 onto the underlying phenylpropanoid biosynthetic pathway structure. Viewed through the lens of the phenylpropanoid biosynthetic pathway [31,32], the organ-specific enrichment patterns reported in Section 3.4 resolve into a coherent pattern of pathway-branch-specific diversion. Within the core flavonoid branch (downstream of chalcone synthase), enrichment in petiole glands increased progressively toward the most terminal biosynthetic steps: flavonols and flavones (35 of 40 differential compounds highest in glands), through dihydroflavonols and flavanols (3/3 and 5/5, respectively), to proanthocyanidins—the final committed products of the flavan-3-ol branch—all 8 of which were exclusive to petiole glands. This gradient suggests that petiole glands function preferentially as a terminal accumulation site for flavonoid pathway end-products rather than uniformly upregulating flavonoid metabolism as a whole. In contrast, the Salicaceae-specific salicinoid/benzenoid branch—a parallel phenylpropanoid-derived pathway distinct from the core flavonoid branch [6]—showed the opposite organ distribution: salirepin, idesin, and demethyl coniferin, all peaked in leaves or shoot tips rather than petiole glands (Figure 6; Section 3.6). This divergence indicates that *I. polycarpa* deploys at least two structurally and biosynthetically distinct classes of phenylpropanoid-derived defense-associated compounds with an organ-specific division of labor. Constitutive salicinoid defenses are distributed broadly across photosynthetic and meristematic tissue, whereas terminal flavonoid/tannin pathway products are concentrated in the petiole gland. A parallel, independent pattern was observed for the tryptophan-derived alkaloid pathway, in which 60% of differential alkaloids peaked in petiole glands, consistent with the nominal (though not multiple-testing-corrected) enrichment of tryptophan metabolism identified by KEGG mapping in both organ comparisons (Section 3.4).

### 4.3. The Tannin and Alkaloid Profile of Petiole Glands Is Consistent with a Candidate Chemical Defense Function

Compositional evidence for a potential defensive role comes from the co-accumulation of two compound classes with well-documented anti-herbivore activity in other plant systems, exclusively or predominantly in petiole glands. First, all 8 proanthocyanidins detected in the study—procyanidins A2, B1–B4, C1, C2, A3—were putatively identified (Level B annotation, spectral matching only) as exclusive to petiole glands. Proanthocyanidins (condensed tannins) deter herbivores by binding and precipitating dietary proteins and digestive enzymes in insect midguts, reducing the nutritional value of ingested tissue [28]. In Populus, a close Salicaceae relative, salicylic acid signaling directly activates condensed tannin biosynthesis in response to fungal infection and herbivore attack [8,33], and poplar proanthocyanidin levels are positively correlated with resistance to phytophagous insects [34], consistent with broader evidence that condensed tannin accumulation in Salicaceae foliage tracks herbivore performance outcomes [35]. The exclusive concentration of the complete proanthocyanidin complement of *I. polycarpa* in petiole glands—absent from the far larger leaf and shoot-tip organs—is consistent with the gland being a major site of condensed tannin accumulation in this species, pending confirmation of these putative annotations with authentic standards.

Second, 15 of 25 differential alkaloids (60%) were most abundant in petiole glands. Alkaloids are among the most chemically diverse and ecologically effective direct-defense compounds in plants, acting through neurotoxicity, enzyme inhibition, and feeding deterrence against a wide range of herbivorous insects [36]. The structural diversity of the alkaloid complement in *I. polycarpa* petiole glands—spanning indole-type, quinoline-type, and phenylamine alkaloids—raises the possibility of multiple defense-related mechanisms co-occurring within a single organ, a hypothesis that awaits functional testing. Notably, tryptophan metabolism—the biosynthetic precursor pathway for indole alkaloids—was nominally the top-ranked KEGG pathway in both organ comparisons (Figure S2), though, as noted in Section 3.4, this did not reach significance after multiple-testing correction and should not be regarded as independent statistical confirmation. It is reported here only as a descriptive observation directionally consistent with the compositional alkaloid signal. The co-occurrence of high-tannin and high-alkaloid profiles in the same organ is well-established in highly defended plant tissues and may represent synergistic toxicity to herbivores [28].

The compositional pattern of the phenolic acid complement is consistent with, though does not confirm, a defensive role. The exclusive presence of galloylated phenolic acid derivatives in petiole glands—including 3,4-digalloylshikimic acid—is consistent with hydrolyzable tannin biosynthesis. Hydrolyzable tannins, like condensed tannins, serve as anti-herbivory defenses and are particularly effective against generalist insect herbivores [28]. Finally, the Salicaceae-specific phenolic glycosides present in *I. polycarpa*—salirepin, idesin, and structurally related compounds—are homologous to the salicinoid defenses of Populus and Salix [6,7]. Feistel et al. [20] demonstrated that *I. polycarpa* leaf phenolics are toxic to lepidopteran larvae, directly linking the Salicaceae-type phenolic chemistry of this species to herbivore defense. Although these salicylate-related compounds peaked in leaves rather than petiole glands in our data, the enrichment of structurally diverse phenolic acids in glands (57 of 107 differential phenolic acids) indicates that the gland is an important additional site of phenylpropanoid accumulation.

### 4.4. Gland-Exclusive Bioactive Flavonoids and Their Potential Significance

The putative (Level B, database-matched) exclusive accumulation of kaempferide, kaempferitrin, and tectoridin in petiole glands is notable for both ecological and applied reasons, pending verification with authentic standards. From an ecological perspective, kaempferol-derived flavonoids are known inducers of resistance against herbivores and pathogens in multiple plant families [25]. Their organ-specific concentration in this externally visible glandular structure is consistent with a candidate contact-defense function, though whether these flavonoids are secreted to the gland surface or retained intracellularly remains to be confirmed (Section 4.4). From an applied perspective, kaempferide has documented antioxidant and other pharmacological activities [24]; kaempferitrin shows anti-inflammatory activity relevant to autoimmune conditions [26]; and tectoridin exhibits strong in vitro antioxidant capacity [27]. We note that these particular pharmacological activities are not themselves evidence of a role in plant defense; the candidate defensive relevance of these compounds rests on the broader, class-level evidence that kaempferol-derived flavonoids induce resistance against herbivores and pathogens [25], rather than on direct functional evidence for kaempferide, kaempferitrin, or tectoridin individually. In kenaf (*Hibiscus cannabinus*), kaempferitrin accumulates at 17–23 µg·mg^−1^ dry weight (DW) in leaves [37], making it one of the richer plant sources known. Beyond its applied potential, this accumulation pattern reinforces the same contact-defense hypothesis discussed above, whether mediated by surface secretion or subsurface accumulation. The present putative identification of kaempferitrin as a petiole gland-exclusive compound in *I. polycarpa* suggests petiole glands as a candidate source for this bioactive compound; standard-based confirmation, quantitative determination, and yield assessment would be logical next steps.

A critical caveat for interpreting the ecological relevance of the gland-enriched metabolite profile is that our metabolomic data were obtained from whole-gland tissue extracts, not from collected gland exudates. Consequently, we cannot distinguish whether the compounds detected are actively secreted onto the gland surface (where they could directly contact visiting insects) or are predominantly stored intracellularly (requiring tissue damage to become bioavailable). This distinction matters for ecological function: surface-localized compounds are more likely to serve as contact deterrents or direct toxins, while intracellular metabolites might function as constitutive reserves that are deployed upon herbivore-induced tissue rupture. The exclusive or enriched occurrence of proanthocyanidins, alkaloids, and galloylated phenolic acids in gland tissue is consistent with either scenario. Histochemical staining (e.g., with vanillin-HCl for proanthocyanidins) and transmission electron microscopy would be required to resolve the subcellular distribution of these compounds. Both approaches have already been demonstrated as feasible in preliminary work by our group. Similarly, collection and chemical analysis of surface washings or gland exudates would help establish whether these metabolites are present in the secretory product itself. Until such data are obtained, the defensive hypotheses advanced here must be regarded as speculative with respect to the precise mode of action.

### 4.5. Contextualizing I. polycarpa Petiole Glands Within Salicaceae Chemical Ecology

The morphological and chemical features described here raise the possibility that *I. polycarpa* petiole glands are structurally and chemically analogous, and potentially functionally homologous, to EFNs or secretory glands reported in other Salicaceae and allied families. Petiolar EFNs in Passiflora recruit protective ants and simultaneously accumulate chemical toxins [2,3]; poplar bud resin glands secrete surface phenolics with antimicrobial and antiherbivore activities [38]. The present data show that, among the three organs examined, petiole glands accumulate the most diverse and concentrated array of metabolite classes previously associated with plant defense in other species—including proanthocyanidins, alkaloids, and galloyl tannins, compound classes not detected in leaves or shoot tips under the analytical conditions of this study (Section 2.4).

A key question raised by the detection of ant visitation in companion field observations [15] is whether the gland metabolite profile reported here is primarily relevant to ant attraction (indirect defense) or to herbivore deterrence (direct defense). These two functions are not mutually exclusive but are expected to involve distinct chemical classes: ant attraction is typically mediated by soluble sugars, amino acids, and volatile organic compounds in nectar or surface exudates [2,3], whereas the metabolites we found to be enriched or exclusive in petiole glands—proanthocyanidins, alkaloids, galloylated phenolic acids, and flavonoid glycosides—are predominantly non-volatile secondary compounds with well-documented anti-herbivore activities in other plant systems [25,28,36]. This compositional bias does not exclude an indirect-defense function; EFNs can produce nectar that contains both attractants and defensive metabolites [3]. However, our metabolomics platform was optimized for non-volatile polar metabolites and did not cover sugars or volatiles systematically, so our data are better suited to inform the direct-defense hypothesis than to assess ant-attractant chemistry. We therefore suggest that the petiole glands of *I. polycarpa* may serve a dual function. The secondary metabolite profile reported here supports a role in chemical defense, whereas the ant visitation observed in the field [15] could be mediated by separate nectar components (e.g., sugars) or volatile cues not captured by the present analysis. Targeted analysis of gland exudate sugars and volatiles, combined with behavioral assays, would be required to resolve the relative contributions of direct and indirect defense in this system.

Whether these glands function primarily through direct chemical defense (as constitutive toxin reservoirs), indirect defense (by attracting mutualistic insects), or both, remains to be determined and represents the central question for future work. Field observations from a companion study further document ant visitation and active liquid secretion at these glandular structures [15], consistent with a candidate indirect-defense (EFN-like) function; however, as in that study, whether this translates into measurable herbivore deterrence remains untested. The compositional evidence presented throughout this Discussion is necessarily correlative: metabolite enrichment patterns indicate where defensive compounds accumulate, but cannot by themselves establish that they function in defense. Confirming this would require herbivore bioassays or induction experiments, neither of which was performed here. Four specific directions would directly address this gap. First, histochemical and electron microscopic characterization of petiole gland ultrastructure could determine whether metabolites are stored intracellularly or secreted onto the surface; this approach has already been demonstrated as feasible by our group using safranin-fast green sectioning and awaits application to batch-matched material. Second, collection and chemical analysis of any gland exudate would clarify whether these compounds are present in the secretory product. Third, herbivore bioassays using gland tissue extracts could test the anti-feeding or toxic activity of the gland-exclusive compounds. Fourth, induction experiments could test whether the gland metabolite profile changes in response to herbivore attack or mechanical damage, which would confirm an active defensive role. Therefore, the ecological interpretations proposed here should be regarded as hypotheses generated from metabolomic evidence rather than experimentally validated functions.

### 4.6. Limitations

Several aspects of the present study design should be noted as limitations. First, each of the three biological replicates per organ was pooled from five individual trees rather than representing a single tree or branch (Section 2.1); this design reduces noise from inter-individual variation and is consistent with the approach used in the companion study [21], but it does not permit assessment of tree-to-tree metabolic variability. Future studies with single-tree or single-branch replication would be needed to resolve this source of variance. Second, because petiole glands were pooled across multiple petioles for extraction, we did not perform paired analysis of the specific leaves from which the sampled glands originated. Such a paired design would allow direct assessment of whether gland-exclusive metabolites are locally concentrated relative to their subtending leaf, as opposed to reflecting broader inter-leaf variability; this represents a logical next step for future work. Third, sampling was restricted to the mid-canopy region, and although this was intended to control for known effects of canopy position on secondary metabolite accumulation [22,23], the organ-specific patterns reported here should be interpreted as characteristic of mid-canopy tissue and may not generalize to upper- or lower-canopy positions.

## 5. Conclusions

Petiole glands of *Idesia polycarpa* represent secretory structures with a secondary metabolite profile that is compositionally distinct from that of leaves and shoot tips of the same plant. This study provides the first systematic metabolomic characterization of *I. polycarpa* petiole glands using UPLC-MS/MS-based widely targeted metabolomics. A total of 700 metabolites were detected in petiole glands—the highest number among the three organs examined—including 39 metabolites detected exclusively in this organ, among them diverse flavonoids, proanthocyanidins, and phenolic acid derivatives. Petiole glands showed preferential accumulation of several secondary metabolite classes, including tannins, flavonoids, alkaloids, and phenolic acids, with a large proportion of differential metabolites reaching their highest relative abundance in glands. Several gland-exclusive flavonoids, including kaempferide, kaempferitrin, and tectoridin, were identified as candidate bioactive compounds, pending confirmation with authentic standards. These compositional patterns suggest a possible role for petiole glands in herbivore defense and other ecological interactions, though functional validation through feeding assays, induction experiments, and other biological approaches will be necessary to confirm these hypotheses. Overall, this study establishes a metabolomic framework for understanding glandular specialization in *I. polycarpa* and provides a resource for future investigations of plant secondary metabolism and gland-associated bioactive compounds.

## Figures and Tables

**Table 1:** Metabolite categories and numbers identified across three organs of *I. polycarpa*.

Metabolite Category	Total	Leaves	Shoot Tips	Petiole Glands
Phenolic acids	130	115	109	127
Flavonoids (subtotal)	120	99	106	117
—Flavones	35	33	34	33
—Flavonols	56	44	48	55
—Isoflavones	11	10	10	11
—Others (flavanols, anthocyanins, chalcones, dihydroflavonols, dihydroflavones, flavonoid carbonosides)	18	12	14	18
Tannins (incl. proanthocyanidins)	13	5	4	12
Lignins and coumarins	25	24	25	23
Alkaloids	29	28	27	26
Terpenoids	27	26	24	24
Lipids	127	115	119	124
Amino acids and derivatives	70	64	69	64
Organic acids	58	57	58	57
Nucleotides and derivatives	40	38	40	40
Miscellaneous (vitamins, sugars, quinones, etc.)	89	86	86	86
Total metabolites detected	728	657	667	700
Organ-exclusive metabolites	46	4	3	39
Shared by exactly two organs	68	---	---	---
Shared among all three organs	614	614	614	614

Note: Organ-level totals for metabolite classes were counted from the UPLC–MS/MS quantitative matrix (Data S1). Subtotals for flavonoid subclasses are not available separately by organ. “---” indicates not separately quantified. Of the 728 total metabolites, 46 were exclusive to a single organ, 68 were shared by exactly two organs, and 614 were shared by all three organs (46 + 68 + 614 = 728). Leaf and shoot-tip biological samples originate from the single collection and analytical batch described in the companion study [21].

**Table 2:** Differential metabolite classes enriched in petiole glands (C) versus leaves (A) and shoot tips (B).

Metabolite Class	Diff. vs. A (*n*)	Up in C vs. A, *n* (%)	Diff. vs. B (*n*)	Up in C vs. B, *n* (%)	Highest in C (*n*/Total)
Flavonoids	91	72 (79%)	69	58 (84%)	72/100 (72%)
Phenolic acids	85	55 (65%)	65	46 (71%)	57/107 (53%)
Lipids	90	84 (93%)	78	56 (72%)	76/105 (72%)
Tannins	9	8 (89%)	13	10 (77%)	10/13 (77%)
Alkaloids	19	14 (74%)	16	11 (69%)	15/25 (60%)
Lignins/coumarins	13	8 (62%)	16	11 (69%)	13/21 (62%)

Note: A = leaves; B = shoot tips; C = petiole glands. Overall, 441 differential metabolites were identified between leaves and petiole glands (A vs. C), and 395 between shoot tips and petiole glands (B vs. C) (see Section 3.3). “Diff.” = number of differential metabolites of that class identified in the respective pairwise comparison. “Highest in C” = Number of metabolites within that class (pooled and deduplicated across both comparisons) that reached their peak mean relative abundance in petiole glands compared to the other two organs.

**Table 3:** Putatively annotated metabolites detected exclusively in *I. polycarpa* petiole glands and their reported bioactivities.

Compound	Class	Reported Bioactivities (Reference)	Confidence Level
Kaempferide	Flavonol	Antioxidant and anti-inflammatory activities have been reported for kaempferide [24]; kaempferol-type flavonoids are documented herbivore-resistance inducers [25].	Level B (MSI Level 3, putatively characterized compound class; no authentic standard)
Kaempferitrin	Flavonol glycoside	Flavonoid glycoside within the same defense-associated class as kaempferide; also reported to have anti-inflammatory activity [26]	Level B (MSI Level 3, putatively characterized compound class; no authentic standard)
Tectoridin	Isoflavone glycoside	Antioxidant [27]; isoflavonoid within a class broadly implicated in plant chemical defense	Level B (MSI Level 3, putatively characterized compound class; no authentic standard)
Dihydrokaempferide	Dihydroflavonol	Biological activity remains poorly characterized.	Level B (MSI Level 3, putatively characterized compound class; no authentic standard)
Procyanidins A2, B1–B4, C1, C2, A3	Proanthocyanidins	Protein-precipitating anti-herbivore tannins [28]	Level B (MSI Level 3, putatively characterized compound class; no authentic standard)
3,4-Digalloylshikimic acid	Galloyl ester	Hydrolyzable tannin precursor; biological activity remains poorly characterized.	Level B (MSI Level 3, putatively characterized compound class; no authentic standard)
Caffeoylnicotinoyl tartaric acid	Hydroxycinnamic deriv.	Biological activity remains poorly characterized.	Level B (MSI Level 3, putatively characterized compound class; no authentic standard)
Hederagenin 3-O-Glc(1→2)Glc(1→4)Ara	Triterpenoid saponin	Biological activity remains poorly characterized.	Level B (MSI Level 3, putatively characterized compound class; no authentic standard)
LysoPC 12:0, 19:0, 20:0; LysoPE 16:1, 20:5	Lysophospholipids	Membrane lipid components; ecological role in petiole glands not characterized.	Level B (MSI Level 3, putatively characterized compound class; no authentic standard)

Note: All compounds listed were identified at Level B according to Metware’s classification (matching of precursor ion Q1, fragment ion Q3, RT, DP, and CE only; no co-injected authentic standards), corresponding approximately to MSI Level 3 (putatively characterized compound class). These annotations should therefore be regarded as provisional pending confirmation with authentic standards. Lyso PC 19:0 is an odd-chain lipid species that is atypical in plant tissue and its annotation is particularly provisional.

## Data Availability

The metabolomics dataset supporting the conclusions of this article is provided in the Supplementary Materials (Data S1). Additional data are available from the corresponding authors upon reasonable request.

## Supplementary Materials

The following supporting information can be downloaded at: https://www.mdpi.com/article/10.3390/metabo16090615/s1, Figure S1: Permutation test results (200 permutations) for the OPLS-DA models discriminating petiole glands from leaves and shoot tips; Figure S2: KEGG pathway mapping of differential metabolites between petiole glands and (A) leaves or (B) shoot tips; Table S1: KEGG pathway mapping of differential metabolites between petiole glands and leaves (A VS C) or shoot tips (B vs. C), corresponding to Figure S2; Table S2: Relative peak areas of key shared functional metabolites across the three organs (mean ± SD; *n* = 3 unless noted); Data S1: Full UPLC–MS/MS metabolite quantification matrix (728 metabolites × 3 organs × 3 biological replicates).

## Abbreviations

The following abbreviations are used in this manuscript:

ANOVA	analysis of variance
APG	Angiosperm Phylogeny Group
EFN	extrafloral nectary
HSD	honestly significant difference
KEGG	Kyoto Encyclopedia of Genes and Genomes
MWDB	Metware Database
OPLS-DA	orthogonal partial least squares discriminant analysis
PCA	principal component analysis
QC	quality control
UPLC-MS/MS	ultra-performance liquid chromatography-tandem mass spectrometry
VIP	variable importance in projection
