# Supplementary material for: Metabolomic Characterization Reveals Organ-Specific Accumulation of Secondary Metabolites in Petiole Glands of Idesia polycarpa
Source: Metabolites. 2026 Aug 27;16(9):615. doi: 10.3390/metabo16090615 (PMC13609106; doi:10.3390/metabo16090615)
Supplement: Supplementary file 1 [file metabolites-16-00615-s001.zip › Supplementary_Materials.pdf]

# Supplementary Materials

Metabolomic Characterization Reveals Organ-Specific Accumulation of Secondary Metabolites in Petiole Glands of *Idesia polycarpa*

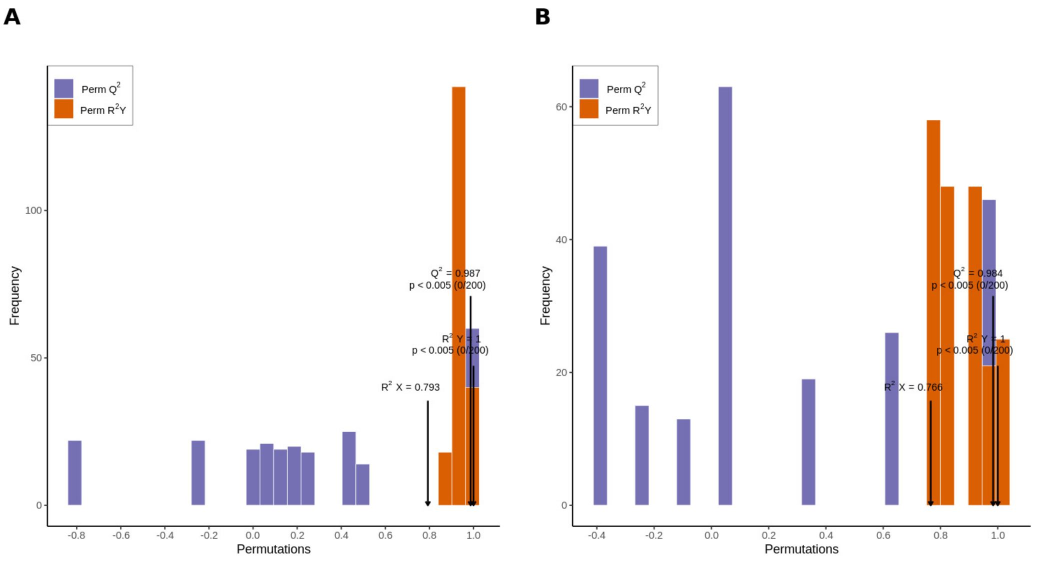


**Figure S1.** Permutation test results (200 permutations) for the OPLS-DA models discriminating petiole glands from leaves and shoot tips. (A) Leaves vs petiole glands (A vs C): $R^2X$ = 0.793, $R^2Y$ = 1.00, $Q^2$ = 0.987; $p$ < 0.005 for both $R^2Y$ and $Q^2$ (0/200 permutations exceeded the original model). (B) Shoot tips vs petiole glands (B vs C): $R^2X$ = 0.766, $R^2Y$ = 1.00, $Q^2$ = 0.984; $p$ < 0.005 for both $R^2Y$ and $Q^2$ (0/200 permutations exceeded the original model). Purple bars indicate the distribution of $Q^2$ values and orange bars indicate the distribution of $R^2Y$ values obtained from 200 permutations of the class labels; arrows indicate the $R^2X$, $R^2Y$, and $Q^2$ values of the original (non-permuted) model.

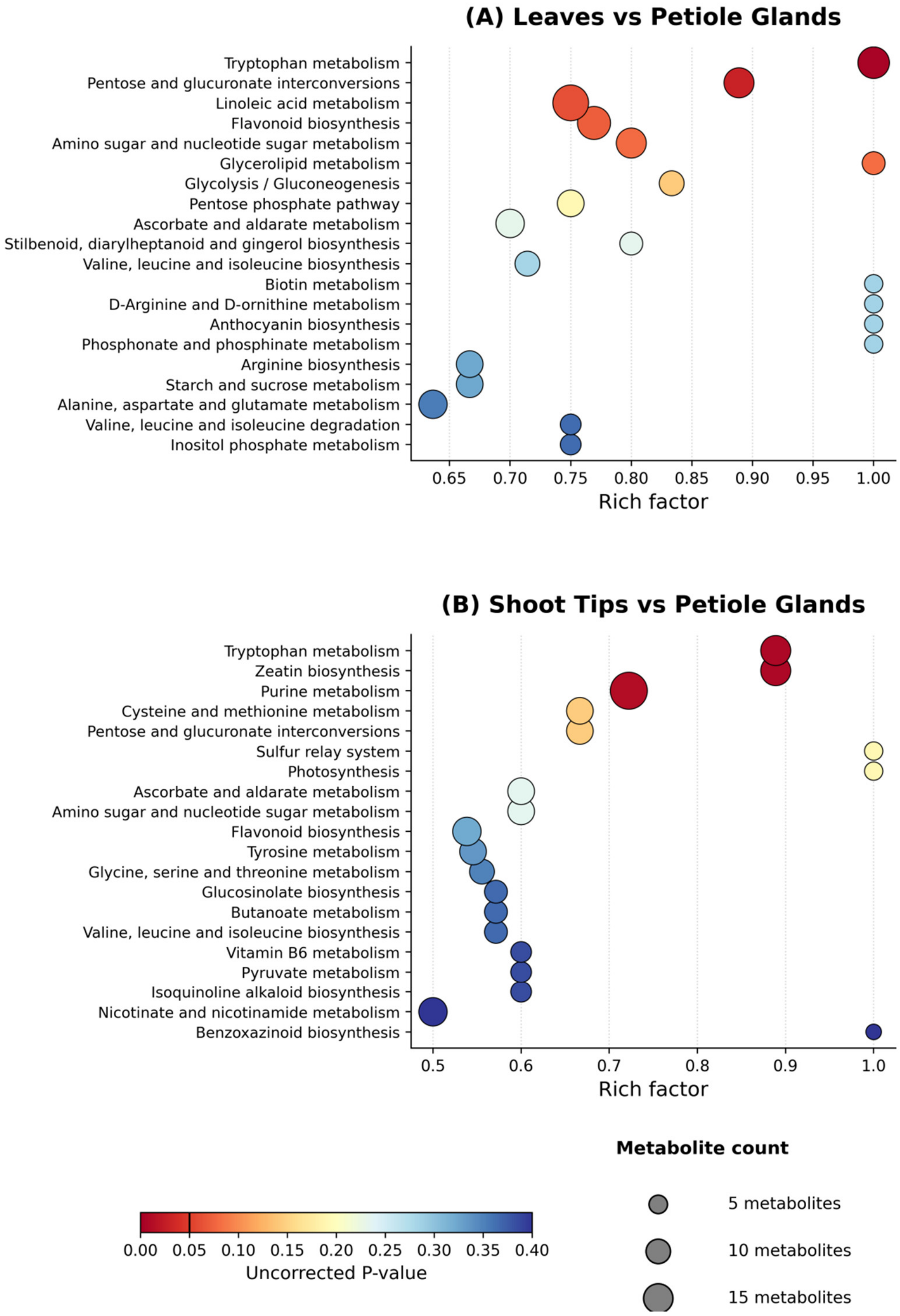


**Figure S2.** KEGG pathway mapping of differential metabolites between petiole glands and (A) leaves or (B) shoot tips. Bubble size indicates the number of KEGG-annotated differential metabolites in each pathway; color indicates the uncorrected hypergeometric *p*-value ($p$ = 0.05 marked on color bar). No pathway remained significant after Benjamini – Hochberg correction (Table S1), likely reflecting the limited number of KEGG-annotated compounds among the gland-enriched metabolite classes central to this study (e.g., tannins, 0/13 annotated; see Section 4.1). Tryptophan metabolism was the top-ranked pathway in both comparisons (uncorrected $p < 0.01$), consistent with the indole-alkaloid enrichment discussed in Section 4.2.

**Table S1.** KEGG pathway mapping of differential metabolites between petiole glands and leaves (A vs C) or shoot tips (B vs C), corresponding to Figure S2. Metabolite counts refer to KEGG-annotated compounds only (260 of 728 total detected metabolites, 36%); pathways are ranked by uncorrected P-value within each comparison (top 20 shown, excluding the non-specific "Metabolic pathways" and "Biosynthesis of secondary metabolites" categories). No pathway remained significant after Benjamini-Hochberg correction.

| **Comparison** | **Pathway** | **KEGG ID** | **Metabolites (diff./background)** | **Rich Factor** | ***p*-value** | **Corrected *p*-value (BH)** |
|---|---|---|---|---|---|---|
| A vs C (Leaves vs Petiole Glands) | Tryptophan metabolism | ko00380 | 9/9 | 1.00 | 0.003 | 0.246 |
| | Pentose and glucuronate interconversions | ko00040 | 8/9 | 0.89 | 0.029 | 1.000 |
| | Linoleic acid metabolism | ko00591 | 12/16 | 0.75 | 0.062 | 1.000 |
| | Flavonoid biosynthesis | ko00941 | 10/13 | 0.77 | 0.071 | 1.000 |
| | Amino sugar and nucleotide sugar metabolism | ko00520 | 8/10 | 0.80 | 0.079 | 1.000 |
| | Glycerolipid metabolism | ko00561 | 4/4 | 1.00 | 0.080 | 1.000 |
| | Glycolysis / Gluconeogenesis | ko00010 | 5/6 | 0.83 | 0.142 | 1.000 |
| | Pentose phosphate pathway | ko00030 | 6/8 | 0.75 | 0.191 | 1.000 |
| | Ascorbate and aldarate metabolism | ko00053 | 7/10 | 0.70 | 0.230 | 1.000 |
| | Stilbenoid, diarylheptanoid and gingerol biosynthesis | ko00945 | 4/5 | 0.80 | 0.231 | 1.000 |
| | Valine, leucine and isoleucine biosynthesis | ko00290 | 5/7 | 0.71 | 0.284 | 1.000 |
| | Biotin metabolism | ko00780 | 2/2 | 1.00 | 0.285 | 1.000 |
| | D-Arginine and D-ornithine metabolism | ko00472 | 2/2 | 1.00 | 0.285 | 1.000 |
| | Anthocyanin biosynthesis | ko00942 | 2/2 | 1.00 | 0.285 | 1.000 |
| | Phosphonate and phosphinate metabolism | ko00440 | 2/2 | 1.00 | 0.285 | 1.000 |
| | Arginine biosynthesis | ko00220 | 6/9 | 0.67 | 0.323 | 1.000 |
| | Starch and sucrose metabolism | ko00500 | 6/9 | 0.67 | 0.323 | 1.000 |
| | Alanine, aspartate and glutamate metabolism | ko00250 | 7/11 | 0.64 | 0.354 | 1.000 |

| | | | | | | |
|---|---|---|---|---|---|---|
| | Valine, leucine and isoleucine degradation | ko00280 | 3/4 | 0.75 | 0.365 | 1.000 |
| | Inositol phosphate metabolism | ko00562 | 3/4 | 0.75 | 0.365 | 1.000 |
| B vs C (Shoot Tips vs Petiole Glands) | Tryptophan metabolism | ko00380 | 8/9 | 0.89 | 0.007 | 0.481 |
| | Zeatin biosynthesis | ko00908 | 8/9 | 0.89 | 0.007 | 0.481 |
| | Purine metabolism | ko00230 | 13/18 | 0.72 | 0.012 | 0.835 |
| | Cysteine and methionine metabolism | ko00270 | 6/9 | 0.67 | 0.144 | 1.000 |
| | Pentose and glucuronate interconversions | ko00040 | 6/9 | 0.67 | 0.144 | 1.000 |
| | Sulfur relay system | ko04122 | 2/2 | 1.00 | 0.191 | 1.000 |
| | Photosynthesis | ko00195 | 2/2 | 1.00 | 0.191 | 1.000 |
| | Ascorbate and aldarate metabolism | ko00053 | 6/10 | 0.60 | 0.233 | 1.000 |
| | Amino sugar and nucleotide sugar metabolism | ko00520 | 6/10 | 0.60 | 0.233 | 1.000 |
| | Flavonoid biosynthesis | ko00941 | 7/13 | 0.54 | 0.321 | 1.000 |
| | Tyrosine metabolism | ko00350 | 6/11 | 0.55 | 0.334 | 1.000 |
| | Glycine, serine and threonine metabolism | ko00260 | 5/9 | 0.56 | 0.349 | 1.000 |
| | Glucosinolate biosynthesis | ko00966 | 4/7 | 0.57 | 0.366 | 1.000 |
| | Butanoate metabolism | ko00650 | 4/7 | 0.57 | 0.366 | 1.000 |
| | Valine, leucine and isoleucine biosynthesis | ko00290 | 4/7 | 0.57 | 0.366 | 1.000 |
| | Vitamin B6 metabolism | ko00750 | 3/5 | 0.60 | 0.385 | 1.000 |
| | Pyruvate metabolism | ko00620 | 3/5 | 0.60 | 0.385 | 1.000 |
| | Isoquinoline alkaloid biosynthesis | ko00950 | 3/5 | 0.60 | 0.385 | 1.000 |
| | Nicotinate and nicotinamide metabolism | ko00760 | 7/14 | 0.50 | 0.417 | 1.000 |
| | Benzoxazinoid biosynthesis | ko00402 | 1/1 | 1.00 | 0.438 | 1.000 |

**Table S2.** Relative peak areas of key shared functional metabolites across the three organs (mean ± SD; *n* = 3 unless noted).

| Compound | Class | Leaves (A) | Shoot Tips (B) | Petiole Glands (C) |
|---|---|---|---|---|
| Pyrocatechol (Catechol) | Phenolic acid | 25,037,667 ± 6,772,683$^{a}$ | 11,767,200 ± 6,660,503$^{ab}$ | 74,995 ± 23,784$^{b}$ |
| Salirepin | Phenolic glycoside | 815,420 ± 22,723$^{a}$ | 324,022 ± 284,928$^{b}$ | 604,830 ± 100,797$^{ab}$ |
| Idesin | Phenolic glycoside | 33,871,667 ± 1,091,770$^{a}$ | 16,102,533 ± 15,557,681$^{a}$ | 26,737,333 ± 5,305,262$^{a}$ |
| Demethyl coniferin | Phenolic acid | 127,407 ± 25,222$^{b}$ | 312,655 ± 126,211$^{a}$ (n=2) | 207,977 ± 27,281$^{ab}$ |
| Idesin salicylate | Phenolic glycoside | 9,497,400 ± 1,185,079$^{a}$ | 54,724,467 ± 73,911,415$^{a}$ | 3,207,200 ± 3,231,145$^{a}$ |

Note: Within each row, values sharing a superscript letter are not significantly different (one-way ANOVA with Tukey's HSD post hoc test, $\alpha = 0.05$); see main text for exact p-values. For demethyl coniferin, the shoot-tip value is based on $n = 2$ biological replicates, as one replicate fell below the detection limit and was excluded from the mean, SD, and statistical test. Kaempferide, kaempferitrin, tectoridin, and all 8 proanthocyanidins were exclusive to petiole glands and are therefore not listed here. Idesin salicylate showed disproportionately high inter-individual variability in shoot tips (coefficient of variation ≈ 135%) relative to leaves and petiole glands; several other phenolic compounds in this table show a similar pattern (see Section 3.6).

**Data S1.** Full UPLC－MS/MS metabolite quantification matrix (728 metabolites × 3 organs × 3 biological replicates). Values in columns A1-C3 represent raw chromatographic peak areas (arbitrary units) for each biological replicate (A: leaves; B: shoot tips; C: petiole glands), as quantified by MRM on the UPLC-MS/MS platform (see Section 2.3 of the main text). The “Level” column indicates metabolite identification confidence (A/B), per Metware/MWDB identification criteria (see Section 2.3). The “kegg_map” column lists KEGG pathway identifiers (ko numbers) annotated for each metabolite, where available.
